# Perceptions of extended-release naltrexone, methadone, and buprenorphine treatments following release from jail

**DOI:** 10.1186/s13722-019-0166-0

**Published:** 2019-10-01

**Authors:** Melissa Velasquez, Mara Flannery, Ryan Badolato, Alexandria Vittitow, Ryan D. McDonald, Babak Tofighi, Ann R. Garment, Jonathan Giftos, Joshua D. Lee

**Affiliations:** 1 Department of Population Health, New York University School of Medicine, 550 First Avenue, New York, NY 10016 USA; 2 Department of Medicine, Division of General Internal Medicine and Clinical Innovation, New York University School of Medicine, 550 First Avenue, New York, NY 10016 USA; 3 Correctional Health Services, New York City Health + Hospitals Corporation, 55 Water Street, New York, NY 10041 USA; 4 New York University School of Medicine, 180 Madison Avenue, 17th Floor, 1714, New York, NY 10016 USA

**Keywords:** Opioid use disorder, Extended-release naltrexone, Incarceration, Reentry

## Abstract

**Background:**

Few studies have documented patient attitudes and experiences with extended-release naltrexone (XR-NTX) opioid relapse prevention in criminal justice settings. This study assessed barriers and facilitators of jail-to-community reentry among adults with opioid use disorder (OUD) treated with XR-NTX, buprenorphine, methadone, and no medications.

**Methods:**

This qualitative study conducted individual interviews with a purposeful and convenience sample of adults with OUD who were recently released from NYC jails. XR-NTX, no medication, and methadone participants were concurrently enrolled in a large randomized controlled trial evaluating XR-NTX vs. a no medication Enhanced Treatment As Usual (ETAU) condition, or enrolled in a non-randomized quasi-experimental methadone maintenance cohort. Buprenorphine participants were referred from NYC jails to a public hospital office-based buprenorphine program and not enrolled in the parent trial. Interviews were audio recorded, transcribed, independently coded by two researchers, and analyzed per a grounded theory approach adapted to the Social Cognitive Theory framework. The research team reviewed transcripts and coding to reach consensus on emergent themes.

**Results:**

N = 33 adults with OUD (28 male, 5 female) completed a single individual interview. Purposeful sampling recruited persons leaving jail on XR-NTX (n = 11), no active medication treatment (n = 9), methadone (n = 9), and buprenorphine (n = 4). Emergent themes were: (1) general satisfaction with XR-NTX’s long-acting antagonist effects and control of cravings; (2) “testing” XR-NTX’s blockade with heroin upon reentry was common; (3) early discontinuation of XR-NTX treatment was most common among persons with high self-efficacy and/or heavy exposure to drug use environments and peers; (4) similar satisfaction regarding effects of methadone and buprenorphine maintenance among retained-in-treatment individuals, alongside general dissatisfaction with daily observed dosing requirements and misinformation and stigmas regarding methadone adverse effects; (5) unstable housing, economic insecurity, and exposure to actively using peers were attributed to early termination of treatment and relapse; (6) individual motivation and willpower as central to long-term opioid abstinence and reentry success.

**Conclusions:**

In the context of more familiar agonist maintenance treatments, XR-NTX relapse prevention during jail-to-community reentry was viewed as a helpful and unique intervention though with important limitations. Commonly described barriers to treatment retention and heroin abstinence included homelessness, economic insecurity, and drug-using peers.

*Trial registration* ClinicalTrials.gov, NCT01999946 (XOR), Registered 03 December 2013, https://clinicaltrials.gov/ct2/show/NCT01999946.

## Background

Increasing access to medications for opioid use disorder (MOUD) among persons involved in the criminal justice system (CJS) is a crucial response to the United States (US) opioid epidemic. Opioid agonist maintenance (methadone and buprenorphine) are effective reentry interventions and are the most commonly prescribed MOUD treatments in the community [1–4]. However, most US correctional facilities, including thousands of local municipal jails, typically do not offer these treatments [5]. Consequentially, the majority of opioid and heroin involved jail detained and sentenced inmates experience opioid detoxification upon jail admission and face high rates of relapse, opioid overdose, and death following release [6, 7]. Extended-release naltrexone (XR-NTX) is a long-acting, injectable, full opioid receptor antagonist. There is substantial evidence supporting the overall effectiveness of XR-NTX in both CJS-involved and general adult participants [8–10]. The use of XR-NTX appears to have recently increased in US jail-to-community reentry programs, though data documenting reentry outcomes are limited [11, 12]. Qualitative research reporting XR-NTX reentry patient attitudes, experiences, and perceived impact on transitioning back to the community is lacking.

Previous studies have identified a number of factors that likely contribute to opioid and other drug and alcohol use after jail, including poverty, income from drug sales, limited family and social support for opioid and other drug and alcohol abstinence or moderation, inadequate access to mental and physical health care, and unstable housing or homelessness [13–16]. Interviews and focus groups conducted among patients with experience on buprenorphine and methadone before, during and after incarceration have documented the profoundly negative experience of forced withdrawal upon incarceration, negative health provider and correctional staff attitudes towards these two opioid agonist treatments, and a common preference not to be physically opioid dependent while in treatment, vs. the overall effectiveness of agonist maintenance in terms of increased function, quality of life, and reduced heroin use [3, 17]. XR-NTX in contrast is an opioid antagonist without physical opioid dependence or risk of withdrawal upon discontinuation. It is not clear, however, how much patients like or prefer active XR-NTX treatment at reentry relative to other treatment options, what motivates treatment retention and consecutive monthly injections when tolerance and withdrawal do not re-enforce adherence, and how individuals adjust when XR-NTX is discontinued.

This qualitative study assessed attitudes towards XR-NTX, methadone, and buprenorphine treatments, and perceived barriers and facilitators of good clinical outcomes during jail-to-community reentry. Individual participant interviews were largely nested in a large randomized controlled trial of XR-NTX vs. no medication treatment among adults leaving NYC jails who were not on or interested in agonist maintenance, *XOR: Extended*-*release naltrexone opioid treatment at jail reentry* (NCT01999946) [18]. A third quasi-experimental observational trial arm of XOR consisted of adults newly initiating methadone maintenance standard of care in jail and at release. In addition, we recruited and interviewed non-XOR patients who had initiated buprenorphine-naloxone maintenance in NYC jail and were referred to our center for continued care.

## Methods

### Study design

We conducted semi-structured, face-to-face, audiotaped interviews with 33 former inmates with OUD recruited from the XOR study and the Bellevue Hospital Center primary care addiction medicine clinic between June 2016 and August 2017. The New York University School of Medicine’s Institutional Review Board approved both the XOR study and the qualitative protocol.

### Setting and population

#### XOR study

*The Extended*-*Release Naltrexone Opioid Treatment at Jail Reentry (XOR) study* [18] is a 24-week, open-label randomized controlled trial examining the effectiveness of 6-months of XR-NTX (N = 85) treatment as opioid relapse prevention at release from jail compared to ‘no medication’, which we refer to as enhanced treatment as usual (ETAU, N = 85). A third, non-randomized, quasi-experimental naturalistic arm of participants who have newly initiated a jail-to-community methadone treatment program (MTP, N = 85) allows for comparisons to a methadone standard-of-care. Methadone detoxification and maintenance are standard of care treatments for all detained and sentenced NYC jail inmates; approximately 10% of all admissions were diagnosed with opioid use disorder in the most recent year (2018). Of that, 73% accessed methadone or buprenorphine maintenance while incarcerated [19]. XOR recruitment takes place at two New York City Department of Corrections (NYC DOC) jail facilities within the Rikers Island jail complex. Upon release from jail, study follow-up visits take place at Bellevue Hospital Center in Manhattan. XOR is one of three trials in the National Institute on Drug Abuse Studies on Medications for Addiction Treatment in Correctional Settings (SOMATICS) collaborative [20], which aims to evaluate XR-NTX, methadone maintenance, and patient navigation interventions for OUD in three large US jail systems (Albuquerque, Baltimore, NYC).

#### Bellevue hospital primary care addiction medicine (buprenorphine) clinic

Since 2006, the Bellevue Hospital primary care addiction medicine program has provided buprenorphine maintenance treatment to all comers and persons released from area jails and prisons. The clinic operates during two half-day sessions per week (Mon and Tues AMs) involving 7+ regular Attending and Addiction Medicine Fellow physician providers. The clinic works directly with the NYC jail opioid treatment program to facilitate buprenorphine transfers the week of jail release and typically receives 0–3 such referrals per week. Treatment and medications are immediately available regardless of insurance status or ability to pay.

Participants were: (a) adults aged 18 or older; (b) formerly incarcerated and released from a NYC jail in the last 24 months; and (c) either actively enrolled in the ongoing XOR study or in Bellevue’s outpatient buprenorphine clinic with a diagnosis of OUD. Persons that met the above inclusion criteria were strategically approached at research or clinic visits and invited to participate in a brief and confidential interview. Study staff obtained informed consent from eligible participants. We used purposeful and convenience sampling to recruit participants on each of the three medications, at various intervals since release from jail, and in response to on-going data coding to achieve data saturation.

### Data collection

After obtaining informed consent, the research staff conducted face-to-face interviews lasting approximately 30 min in a private room at Bellevue Hospital Center’s ambulatory care unit. Participants were compensated $20 for their time and effort. Interviews were audio-recorded, transcribed verbatim, and combined with researcher’s field notes. Medical students, research coordinators, and investigators trained in qualitative research methods conducted the interviews.

### Interviews

The semi-structured interview guide focused on overall reentry trajectories, medication effects and treatment retention, and in iterative fashion, emergent themes from earlier interviews. The guide was derived from the Social Cognitive Theory (SCT) and a prior NYC trial assessing perceptions pertaining to buprenorphine and OUD care among CJS involved participants [3]. SCT describes the influence of personal experiences, the action of peers, and environmental factors on individual health outcomes, which has often been applied to addiction [21]. According to SCT, individual experiences and perceptions regarding self-efficacy, outcome expectations, goals, and structural barriers/facilitators are all important domains relating to behavior change [22]. Our interview questions touched on each of these key determinants of behavior change, and more specifically, how they influenced treatment engagement, retention, and post-incarceration opioid and other drug use.

### Analysis

Interviews were transcribed verbatim, verified for accuracy, entered into Atlas.ti [23], and analyzed line-by-line by two independent coders. Key codes were then discussed in-depth by the study team and consolidated into a codebook using a grounded theory approach adapted to a Social Cognitive Theory framework. Our application of grounded theory allowed reviewers to independently review transcribed interviews to yield key codes, sub-codes, and code ‘clusters’ that were organized into themes. The codebook was continuously refined based on emerging themes and adapted to the Social Cognitive Theory framework to identify topics related to MOUD treatment experiences during reentry. Reviewers ensured inter-coder reliability using the constant comparative method [24, 25] and developing coding schemes following the initial nine interviews; the interview script was then edited to better probe prominent codes and emerging themes topics and subsequent independent coding continued open coding as well as the newly established categories. Although grounded theory is not typically intended to capture data pertaining to the SCT model, data initially organized into relevant themes were compared to the SCT model and demonstrated minimal need for adaption [26, 27]. Discrepancies and ambiguities pertaining to code findings were discussed with senior investigators until consensus was reached. Methodological rigor was ensured by maintaining an audit trail of process and analytic memos, coding books, and periodic debriefing conducted by the qualitative data analysis team. Participants’ responses regarding barriers and facilitators to maintaining abstinence and successful community reentry were organized into 3 areas: (1) Treatment-level, (2) Environment-level, and (3) Patient-level. There was some coding overlap between these three levels. For example, heavy exposure to drug use in the environment could lead to extinguishing treatment early.

## Results

### Participant characteristics

We interviewed 33 participants enrolled from the XOR study (n = 29) and the Bellevue office-based opioid treatment (OBOT) program (n = 4) (Table 1). Most participants were male (n = 28, 85%) and self-reported as African American (45%) or Hispanic (36%). The mean length of time since release from jail was 14 weeks, ranging from just 1 week to 19 months after release. We attempted to recruit participants evenly across the four treatment groups; fewer buprenorphine patients were available during the recruitment period. XOR participants varied in terms of their original medication; several had discontinued XR-NTX or methadone, or crossed-over to a different medication, usually to buprenorphine. Participants receiving buprenorphine treatment were all active in treatment at the time of interviews. We were unable to interview former buprenorphine patients who had subsequently discontinued treatment.

**Table 1:** Demographic characterists

	Mean (range)
Weeks since release	14 (1, 76)
Age	47 (23, 60)

	n (%)
Gender	
Male	28 (85)
Female	5 (15)
Race/ethnicity	
African American	15 (45)
Hispanic	12 (36)
Caucasian	4 (12)
Other	2 (6)
MOUD treatment	
XR-NTX	11 (33)
No medication	9 (27)
Methadone	9 (27)
Buprenorphine	4 (12)

### Treatment-level barriers/facilitators

Nearly all participants had engaged in some form of opioid pharmacotherapy previously, usually methadone. Participants shared current and past treatment experiences, focusing on the medication’s effectiveness or lack thereof to curb cravings and heroin use, as well as the adverse effects of treatment and whether access to delivery of treatment was easy or difficult.

#### Extended release naltrexone (XR-NTX)

Those receiving XR-NTX universally acknowledged they had never heard of XR-NTX or “Vivitrol” before XOR recruitment. Extensive OUD treatment histories were common in both the community and during incarceration. Most were eligible for methadone and/or buprenorphine maintenance during their incarceration through the NYC jail’s opioid treatment program, but instead opted to initially detox and then remain opioid-free while incarcerated and at release. Reasons for not wanting agonist treatment varied. Several expressed their interest to remain opioid-free, not wanting to, “trade one addiction for another,” or be required to attend a methadone treatment program daily. Others feared painful withdrawal symptoms if they decided to discontinue agonist treatment in the community or feared potential side effects often brought on by misinformation and stigma.

In this small sub-sample of XOR XR-NTX participants, the lack of knowledge regarding XR-NTX treatment did not prevent any from receiving a first XR-NTX injection in jail, typically within a week prior to a known release date. Participants overwhelmingly expressed initial skepticism regarding XR-NTX’s effectiveness as an opioid “blocker.”

Following release from jail, around half of participants receiving XR-NTX admitted to using a small amount of heroin within the first 4 weeks upon release, often reporting a desire to “test” whether the medication truly “worked.” One participant expressed his initial reaction to testing the opioid blockade after reentry:

*“I just tried it [heroin], just to experiment, to see if I would feel it or not. But I didn’t. I didn’t. One bag, one, that’s it. I was like nah I’m not gonna waste my money on that. I’m just gonna try to stick to the shot.”* [Participant 21]

Others on XR-NTX who used heroin shortly after release acknowledged having forgot that they had received XR-NTX, or not recalling information about XR-NTX blocking the effects of opioids:

*“I was trying not to [use], but they gave me a [bag] for free, and then I bought another one and it ain’t do nothing to me. But I forgot I had the medicine [XR*-*NTX] in my body…I thought he was selling me a dummy, then I realized oh wait a minute it was working. That’s when I said, you know what, and since then I’m doing good.”* [1]

Some participants reported anticipating that they would feel some physiological effects on XR-NTX similar to agonist treatments. However, most reported forgetting they were on XR-NTX entirely even after multiple injections and expressed skepticism of its effectiveness due to a lack of daily reinforcing opioid effects. As one participant stated:

*“Well I mean unlike methadone it’s like I don’t even acknowledge that I receive the shot or received any type of treatment in terms of the shot. It’s nothing like being on methadone or buprenorphine…those 2 drugs have the potential to be misused tremendously, whereas you know I’ve gotten the shot and I don’t think about it.”* [22]

All participants who used heroin while on XR-NTX shortly after release confirmed that XR-NTX blocked the euphoric effects of illicit opioids. In most cases, confirmation that the blockade worked immediately distinguished additional use by XR-NTX participants. One participant who tested the XR-NTX blockade explained:

*“It’s a waste of time cause you know, I ain’t gonna feel nothin’ cause the doctor even told me you ain’t gonna feel nothin’ and it’s true, I tried it and I didn’t it was true, I ain’t feel nothin’ it. I said it’s a waste of time, I ain’t wastin’ my money on this.“* [27]

Another participant while on XR-NTX did not initially admit to using opioids at a study visit, but after being shown their urine toxicology was positive for heroin explained:

*“She [the Research Coordinator] told me from day one you’re not going to feel it. You know but me with my hard head, you know I have to test the waters you know, I have to test the waters. But it has gotten a lot better, it really has you know. I was dippin’ and dabbin’ now you know and I was like, you know this doesn’t make no sense, you just spending 20, 30, 40 dollars for what, just givin’ the man your money. Cause you don’t feel it.”* [21]

Attempting to overcome the blockade with larger amounts of opioids was not reported; there were no reported opioid overdoses by any XR-NTX participants interviewed. A few participants reported side effects of XR-NTX including headaches, upset stomach, and nausea. These were qualified as tolerable and acceptable and not preventing further treatment.

The attenuation of opioid cravings was a common effect mentioned in interviews by those treated with XR-NTX. Participants lauded this reduction in cravings that made it easier to avoid opioid relapse especially in neighborhoods where access to opioids was readily available. One participant described this reduction in cravings, saying:

*“I mean it [XR*-*NTX] helps because I haven’t been craving for what I used to do and that was heroin. So I mean I’ve been around it since I’ve been home and I was offered it too, and you know I was like nah, I’m alright you know what I’m sayin’. So it is the shot, it helps a lot and yourself. So I don’t have a craving for no opiate.”* [5]

Another participant similarly reflected on less cravings and some positive aspects of being opioid-free on XR-NTX:

*“You know I sleep at night you know and I do take care of my business like I supposed to…the first thing on my mind is not a bag [of heroin] when I wake up in the morning, which it was back in the days when I was using and I didn’t have the shot…but now I can function without it [heroin].”* [18]

One participant, who had previously tried both methadone and buprenorphine, touted the fact that XR-NTX had no potential for misuse. She also reported improved treatment adherence, decreasing the frequency of treatment from daily to monthly:

*“The cravings have definitely lessened and like I said I’m so grateful that I’m on [XR*-*NTX] because probably I’d already have a habit. You know, I don’t have to get up every day to make sure I take medication or get up every day to make sure I go to some program. So it is, in that sense it’s very helpful.”* [3]

Although all participants agreed that XR-NTX lessened or nullified cravings and most conveyed general satisfaction with XR-NTX treatment, some participants had discontinued XR-NTX treatment after an initial 1–3 of 6 planned monthly injections. One participant who received an initial 2 injections (1 pre-release, 1 post-release) was interviewed during the first few weeks post-release while on XR-NTX said:

*“The shot [XR*-*NTX] helps, the shot stops you if you do try to get high. What’s the sense, I’m not feeling anything…I haven’t been craving for what I used to do and that was heroin. So I mean I’ve been around it since I’ve been home…and I was offered too and you know I was like nah, I’m alright”.* [23]

After 2 months, he discontinued study visits and XR-NTX treatment. Eventually research staff made contact with him and conducted follow-up interview months later, when he described stopping XR-NTX and resuming heroin use:

*“Cause the environment I’m around, if I don’t come around, I don’t indulge in things, and so it is, it is the environment… because a person around [you] might be doing it [heroin] you see them high around…will make you say, I’m not gonna go get the shot because I wanna feel like that person, I wanna look like that person, you know what I’m saying. That’s one of the reasons why someone will stop coming to get the shot. And, I mean, that’s the big reason from being around a person who’s getting high, and you’re not upset you took the shot, but you wanna feel how you used to feel, that that past that you miss, you lookin’ at like…it give you thoughts, like, I’m gonna try [heroin], I’m not gonna take the shot this month, know what im sayin’. Cuz it lasts for 30* *days, it really have you thinkin’, like damn, I’m not gonna be high for 30* *days”* [5]

Discontinuing XR-NTX treatment centered on exposure to actively using peers and preferences to adopt lifestyles attributed to active use:

*“The feeling is different [taking heroin on XR*-*NTX]. [The heroin] doesn’t have the same kind of pull, you see. Some people might want the real deal, so that’s one reason people might stop [XR*-*NTX]. But for someone that wants to stop, it’s good.”* [20]

However, not all XR-NTX participants who used opioids shortly after release dropped from treatment. In fact, several participants tested the blockade, acknowledged it worked to subdue or extinguish the reinforcing effects of opioid use, and continued XR-NTX injections through the remainder or majority of the study’s treatment phase. Treatment retention among this small subset of participants who used opioids within the first 4 weeks post-release appeared as good or better than those who used opioids later in treatment (> 4 weeks post-release).

Overall, participants had positive experiences on XR-NTX with few negative observations. Some participants did express apprehension about how they would remain opioid-free once XR-NTX treatment ends. As one participant explained:

*“At one point in time I was dependent on the shot, cause it do works. And when you can’t get it…you go back to doing other things, which you really don’t wanna do.”* [23]

Other participants referred to XR-NTX as a “crutch” or an “insurance policy”. While on XR-NTX participants in general did well to avoid resumption of previous opioid-use. Even if they tested the XR-NTX blockade, most used only small amounts and kept coming back for additional treatment injections suggesting XR-NTX treatment was favorable. When one participant was asked if he would recommend XR-NTX to others with OUD, he stated:

*“When I was taking it [XR*-*NTX]…it works, it works it do[es]. It helps me save money and stops me from getting high. Cause I tried it* – *I tried the drug to see if it works, and it do works. If anybody comes and asks, send ‘em to me. I’ll tell them the shot works”.* [24]

#### Methadone maintenance treatment

Perceptions of methadone varied, as did retention in community methadone treatment post-release. One participant termed methadone, “life-saving,” while others viewed the chronic opioid treatment negatively. Many of the negative viewpoints were based on actual treatment experiences while others appeared heavily influenced by misinformation and stigmas regarding methadone treatment programs (MTP).

One respondent who had never engaged in any MOUD treatment prior to initiating methadone maintenance in jail was extremely satisfied:

*“I want to say [methadone] it saved my life basically, I mean I never been on the methadone program before, I heard about it and always want to try it and I don’t know…I just was serious about the program and I really took everything you know 100%, I just wanted to really get clean and sober this time around you know, and the methadone program really changed my attitude…”* [16]

He went on to discuss how methadone was instrumental in reducing his cravings for heroin, saying:

*“…physically [it] helped with the cravings, I wanna say, and I really have to say that’s due to the methadone, I really think cause um before that I was having cravings like you know three times a day, four times a day you know but now I don’t have any cravings, don’t think about it.“* [16]

Other participants revealed that they accessed methadone treatment in jail only as an “extended detox” to avoid experiencing withdrawal symptoms during incarceration with little intention of following-up in their assigned community methadone program. One methadone participant described his dislike for methadone along with his fear of withdrawal from methadone:

*“… what I didn’t like the most about it was the retaining of the water, the weight gain, the messing of the teeth, the bone hurting…and if I stop taking it withdrawals is unbelievable! So painful. I mean it’s worse than heroin itself. It’s worse than kicking heroin itself. And I didn’t understand it, how is that even helping. Why, how could you do that and say it’s supposed to help us stop when you actually doin’ more physical harm than the drug itself, literally.”* [17]

Similar to this participant, others did not adhere to methadone treatment due to misinformation or common stigmas. In addition to its addictive potential, methadone was perceived to be harmful to a person’s bones and teeth, as well as leading to insomnia and weight gain. Participants reported they were turned off by negative influences and continued opioid use around the methadone clinic, which they believed hindered their recovery:

*“That methadone is no joke. Methadone destroys your body. I never like the program because I used to go to the program and I used to get my meth and I still used to go and get high. People all around there, they get high.”* [8]

*“[I was going to the MTP] on and off because I was trying to overcome my sickness. I ain’t going to lie to you. I didn’t want to tell them that, I just wanted to let it rock out when I was going, just in case I did get sick, I could always run there [to the MTP] and…right after I would go there [MTP] I would go get a bag or two.”* [20]

Many participants described the methadone treatment program daily dosing requirements as intrusive and interfering with other responsibilities (i.e., securing employment, housing, traveling). One participant described methadone using the familiar term, “liquid handcuffs.” A participant expressed his frustrations with the program’s strict attendance policy:

*“It’s like you chained up you have to be there every day, you know you can’t go nowhere, you can’t travel, you can’t do nothing because you got to go to your meth, pick up your meth.”* [14]

#### Buprenorphine maintenance treatment

In contrast to XR-NTX and methadone participants, all four buprenorphine participants were currently active on buprenorphine-naloxone maintenance. Most spoke positively about buprenorphine, were satisfied with their care, and intended to continue treatment. One participant credited buprenorphine treatment for avoiding heroin use post-release:

*“My life is falling back into place little by little and I will give the credit to the medication [buprenorphine] because without it I would be using [heroin].”* [7]

Another participant acknowledged that buprenorphine treatment was crucial in helping him stay on track post-release and work towards achieving his daily goals, stating:

*“Suboxone. That’s the only thing that helps me achieve my daily goals. I set a goal and try to accomplish it. If I didn’t have that [Suboxone], my life would be out of control. If I was on dope, that would be my only goal. I’d have to get that dope, I’m not gonna get sick. Knowing I can take my meds as directed and go about my day makes it all worthwhile.”* [28]

Some described how they often have to negotiate between treatment, probation/parole, employment appointments, and the detrimental impact of scheduling conflicts that occur among those in agonist treatment programs. At least one participant reported that the office-based buprenorphine interfered with other commitments, but felt it was crucial to their health and recovery to make clinic attendance a priority:

*“Today I was supposed to go to work and a probation appointment, but I skipped those to be here. I am dedicated to Suboxone.”* [29]

Access to OBOT programs upon reentry was difficult for some participants due to long waitlists, lack of insurance coverage, and poor clinical care after their initial encounter with program staff. Some generalized their negative views of opioid pharmacotherapy, stating that there was great potential for misuse and diversion.

Other participants had more positive experiences in buprenorphine treatment and stated that they preferred buprenorphine over other MOUD’s. Participants were generally satisfied with the physiological treatment effects, particularly citing that buprenorphine decreased their cravings for opioids. One interviewee was grateful that while on buprenorphine he could sustain a job and did not, “…*have to worry about urges, making lines, [or] leaving early to get to the program.”* [7]

### Environmental-level barriers and facilitators

Participants expressed the importance of having their basic needs met first upon reentry before addressing treatment needs in the community. Difficulties in securing housing and employment were common, and were made more complicated by strained family and personal relationships. Several participants noted either chronic or recent homelessness as a primary barrier to maintaining abstinence and adhering to prescribed treatment. For other participants, securing housing was both a goal and a motivation to avoid relapse, highlighting the integral role of stable housing among NYC opioid users. As one explained:

*“I think home is the biggest thing because when you’re trying to focus on where you’re going to sleep at you can’t really focus on anything else…Cause sometimes it’s like I got the shot [XR*-*NTX], or a magic pill [buprenorphine], like everything is going to be okay. No! You need all those other things in place, mechanisms in place to make this successful.”* [3]

Participants described emotional distress and heroin use linked to homelessness immediately post-release. This was often unanticipated after learning family or friends no longer offered housing supports due to their own misfortunes and instability. Those accessing homeless shelters characterized these facilities as dangerous, stressful and marked by rampant drug and alcohol use and physical and verbal altercations. A participant shared his frustrations with the shelter by comparing it to jail:

*“The shelter is hard, I’m trying to get my things together and save some money to get out as quickly as possible…It’s even worse than the place that I came from [NYC jails].”* [30]

Numerous participants noted the financial strains former inmates face upon release. Some even struggled to find enough money to pay for public transportation to attend treatment programs. In one instance, financial constraints led to food instability and missing a scheduled visit to receive XR-NTX treatment. Another participant sacrificed food in order to attend their buprenorphine clinic appointment. As this participant explained:

*“The soup kitchen is open 10:30 to 12:30… Now I missed lunch, so now I gotta think where am I gonna eat? But that’s ok, I’ll sacrifice that for my meds.”* [28]

Another common barrier discussed throughout interviews was the frequent exposure to neighborhoods and peers where heavy drug and alcohol use is common. One participant described returning to his old neighborhood after some time out-of-state and following discontinuation of XR-NTX:

*“I came back to New York around mid*-*October…[when I relapsed] I was in the same area in Brooklyn…back with my friends who use. The cravings weren’t that bad when I picked back up. I didn’t really feel [the heroin] the first time [in mid*-*October]. By November I was sniffing 2*-*5 bags a day.”* [19]

These neighborhoods were often where participants had previously bought or used heroin. Living in or frequenting these neighborhoods was a major obstacle to abstinence regardless of treatment status. One participant recalled that when avoiding or declining heroin use, he experienced resentment from actively using peers:

*“Environment is very important, because you have a lot of peer pressure. People, places, and things, so being in the wrong area with the wrong people, they don’t wanna see you succeed*. *If you’re not in that particular area, you get a different perspective from other people.****”*** [20]

One ETAU participant who remained heroin-abstinent without receiving any active medication treatment highlighted, “…*the less people you know, the less you can drug*.” *[*8*]* Others maintained abstinence by avoiding prior neighborhoods associated with misuse. Several participants were able to seek support from sober social networks formed by peers and family members, while others did so by participating in mutual help groups.

Employment was a constant challenge. Prior felonies, older age, and homelessness were obstacles to obtaining employment. In one instance, a participant recalled how his employers distrusted him because of his felonies and history of opioid use:

*“What‘s blocking me? Felonies. I got too many felonies. Stops me from getting a job, like I‘ve done custodial maintenance, so I went to the hospital and the manager told me ‘Yo, if I hire you there’s drugs all over the hospital, what if you get busted with drugs? You think I’m gonna trust you?’ So they don’t want to hire you because of the felonies.”* [1]

### Patient-level barriers/facilitators

Participants emphasized the importance of several factors influencing self-efficacy following reentry. Reflections on their prior experiences with active drug use/illicit activities allowed some participants to maintain abstinence, while other individuals attributed such recollections as exacerbating relapse. The majority of participants believed that maintaining sobriety upon release from jail was possible largely through “willpower” and the “motivation” to remain abstinent, despite adverse reentry experiences:

*“Only you yourself will stop…it’s up to you, you have to want it, you have to want this, you have to know deep in your heart that you want to stop. Otherwise you’re never going to be successful. I’m from neighborhoods where everything’s existing, every day I walk out that door it [heroin]’s right there in front of me, but like if you set your mind to it no is no.”* [2]

*“It’s my own motivation. It doesn’t matter where you go, there is drugs and criminal activity everywhere.”* [31]

Others stated they were able to control their urge to use by constantly “keeping busy” and distracting themselves in any way possible. A participant reflected on this approach saying:

*“If my mind occupied and doin’ somethin’, I don’t really indulge. If I’m bored, got a lot of idle time, that’s when I get high.”* [14]

Some participants were motivated by previous periods of sobriety, stating that they would be able to achieve sobriety again despite relapsing in the past. For others, a reluctance to return to their old lifestyle served as the main motivation to remain opioid-free. They highlighted a desire to implement enduring lifestyle changes in order to remain out of jail. One participant commented that he was tired of “…*living life on the hamster wheel.*” Some described avoiding relapse as a life and death situation:

*“I’m extremely motivated [to stay opioid*-*free]. There’s nowhere to turn back. I gotta go forward, if I turn back I’m gonna die.”* [5]

Another participant expressed a similar sentiment about avoiding repeating previous mistakes, while noting an improved self-image as a result of his current sobriety. He shared his satisfaction concerning both his outward appearance and current lifestyle:

*“[I’m] 150% motivated to stay clean. I’m not going back to that. That chapter of my life has closed. I lost too much in that game. I spent my childhood in prison because of it…. I don’t think about any substance to get high on. I’m just good without it. I feel good, nice and clean, I feel good looking at myself in the mirror.”* [30]

Some participants stated no motivation or desire to remain abstinent when interviewed. These individuals reported relapsing immediately upon release. At the time they were interviewed, these participants were generally no longer treatment seeking and off XR-NTX or methadone if previously treated. Of those that managed to or had a strong desire to remain opioid-free, all expressed the necessity of having a source of motivation from self, family, peers, or faith in a higher power.

## Discussion

Participant interviews focusing on XR-NTX suggested a limited understanding of XR-NTX‘s properties and mechanisms of action at baseline and treatment initiation, followed by generally positive and acceptable post-release experiences. To our knowledge, this represents initial and novel qualitative data describing the XR-NTX for OUD corrections-to-community treatment model. Despite learning about XR-NTX for the first time while incarcerated and prior to release, all XR-NTX participants interviewed had readily accepted a first injection. This was consistent with recent publications supporting the feasibility and interest of XR-NTX treatment among criminal justice populations [8, 11, 28]. The possible appeal of opioid-free medication modalities is not exclusive to CJS populations. Two recent non-US qualitative studies documented a general interest and willingness to receive XR-NTX treatment as an alternative to opioid agonist therapies [3, 29, 30]. Overall, active and recent XR-NTX participants noted reduced cravings, were satisfied with a monthly injection as opposed to daily dosing, and credited XR-NTX’s lack of misuse and diversion potential and non-association with negative MOUD treatment stigmas. Two recent qualitative studies concerning MOUD documented parallel XR-NTX attitudes among prescribers and CJS personnel [31, 32].

Although participants were educated at length by study staff about XR-NTX and how it worked to block the effects of opioids, about half admitted using a small amount of heroin to test the blockade shortly after release. Interestingly, immediate post-release opiate use did not appear to drive drop-out. The majority of XR-NTX participants who tested the blockade, described a convincing lack of opioid effects and continued XR-NTX injections as planned.

Discontinuing XR-NTX treatment prior to study treatment end was usually described as intentional. Some described a clear intention to discontinue naltrexone in order to be able to use opioids again, often influenced by exposure to opioid-using environments and peers. Others described a feeling of confidence and ‘cure’ while on XR-NTX, which led to a decision to forgo further treatment given the participant was surviving opioid-free with little apparent difficulty. From a social-cognitive theory perspective, high self-efficacy or a person’s belief in their own abilities is often viewed positively. However, these results indicated a possible reverse effect among XR-NTX participants. High self-efficacy among XR-NTX participants led some participants to discontinue treatment early, believing they were in full control of their abstinence from opioids and that medications were no longer needed. It is unclear whether this high degree of self-efficacy was always present in participants or whether it was inflated due to this most recent period of abstinence from opioids while on XR-NTX. Similar to a recent pilot study [33], there were no reports of XR-NTX participants attempting to override the antagonist blockade with excessive opioid doses, which remains a risk of naltrexone but seemingly not a prevalent or routine event [34].

Participants’ perceptions and experiences varied widely among those in methadone treatment. Methadone was the most frequently used therapy for OUD in jail to avoid withdrawal symptoms in NYC jails. Several methadone participants did not link to community MTP and reported no prior intentions of doing so while incarcerated, despite the jail-based methadone programs goal of referring all maintenance cases to a community MTP. Among those that did enroll in an MTP post-release, there was general satisfaction among those retained in treatment and doing well. Overall, methadone participants were critical of daily attendance requirements and were wary of withdrawal symptoms upon discontinuation. Misinformation and stigma concerning methadone side effects, including dental and musculoskeletal problems, insomnia and weight gain were common. Similar findings have been described in one previous qualitative study [17].

Buprenorphine treatment was viewed as an acceptable and relatively stigma-free form of treatment for OUD. Favorable views of buprenorphine may have been related to the primary care setting at which all the participants received post-release care, as opposed to licensed intensive outpatient drug and alcohol programs [24, 35]. Participants reported less side effects compared to methadone and fewer fears of withdrawal. Most buprenorphine respondents praised the OBOT treatment experience, which included more flexible and extended follow-up intervals following stabilization (i.e., weekly or monthly follow-up visits) compared to daily dosing. Some noted negative past experiences enrolling in outpatient buprenorphine programs including long-waitlists and difficulties with insurance and prescription refills.

Our findings confirmed the extensive barriers that recently incarcerated adults with OUD face when transitioning back to the community. All participants interviewed reported adverse reentry conditions, most commonly homelessness, widespread exposure to drug and alcohol use, unemployment, poverty, and financial instability. Each of these factors were identified as barriers to linkage and retention to community-based treatment, maintaining long-term abstinence, and successful community reentry. These findings are aligned with other recent studies of former inmates leaving jail unprepared and lacking crucial resources [7, 8, 14, 36].

Despite these obstacles, the majority of participants expressed a readiness to change and avoid opioid use after release. Participants frequently mentioned that individual willpower was key to avoiding relapse, versus access to medications. This finding was documented in prior work among formerly incarcerated opioid-involved New Yorkers [3]. Considering the majority of interviewees return to neighborhoods where heroin is easily available and major life stressors are prevalent, the notion that sufficient willpower is a key to avoiding relapse seems logical. However, they seemed to believe individual motivation and willpower as much more important than medication adherence. A clear public health and CJS priority appears to be better informing and motivating individuals towards MOUD access and adherence, as opposed to general, non-specific desires to remain heroin-free.

Study limitations included a convenience sample of participants enrolled in the XOR study and a local NYC primary care buprenorphine program, which may not generalize to other locations or specific reentry paradigms. Nor did this small number of methadone (n = 9) and buprenorphine (n = 4) participants, who were non-randomly and consecutively sampled, represent the much larger annual cohorts of agonist maintenance patients leaving NYC jails (~ 4400 who accessed methadone or buprenorphine maintenance in 2018) [19]. We attempted to conduct interviews evenly across all treatment groups but were unable to recruit many buprenorphine participants, chiefly due to a limited recruitment period. We did not interview individuals following drop-out or discontinuation of buprenorphine treatment, unlike the XOR study patients on XR-NTX or methadone. All interviewers had training in qualitative interviews and analysis. Although prior qualitative experience was quite varied and could impact results and the thoroughness of data depth. Participants were interviewed at various time points after release from jail from 1 week to one-and-a-half years. Understandably, there may have been some recall bias among participants interviewed long after their release date.

## Conclusions

Extended-release naltrexone treatment during jail-to-community reentry was seen by treated participants as a useful post-release relapse prevention option. More commonly-used agonist treatments were similarly beneficial with some drawbacks. Participants described the numerous barriers to treatment retention and heroin abstinence, most notably homelessness, limited finances, and heavy exposure to drug-using peers. Developing better information delivery of and access to medications to treat opioid use disorder in jails with post-incarceration treatment plans in the community is crucial to post-release success.

## Data Availability

Not applicable.

## Abbreviations

CJS: criminal justice system
ETAU: enhanced treatment as usual
MOUD: medications for opioid use disorder
MTP: methadone treatment program
NYC: New York City
NYC DOC: New York City Department of Corrections
OBOT: office based opioid treatment
OUD: opioid use disorder
SCT: social cognitive theory
SOMATICS: *Studies on Medications for Addiction Treatment in Correctional Settings*
US: United States
XOR: *extended*-*release naltrexone opioid treatment at jail reentry* study
XR-NTX: extended-release naltrexone
